# Vitamin D deficiency among apparently healthy adults in northern China: behavioral correlates and an indoor-lifestyle framework

**DOI:** 10.3389/fpubh.2026.1871195

**Published:** 2026-07-08

**Authors:** Jin Tian, Xiaojing Yan, Yongjian Liu, Yongchao Song, Yizhou Ji, Baogen Zhao, Yujing Mi, Xiaolong Li, Liya Jiao, Li Yang, Longmei Tang

**Affiliations:** 1 Hospital Management Innovation Research Center, The First Hospital of Hebei Medical University, Shijiazhuang, Hebei, China; 2 Health Management Center, The First Hospital of Hebei Medical University, Shijiazhuang, Hebei, China; 3 Public Health Department, The First Hospital of Hebei Medical University, Shijiazhuang, Hebei, China; 4 Department of Radiology and Nuclear Medicine, The First Hospital of Hebei Medical University, Shijiazhuang, Hebei, China; 5 Medical Laboratory Center, The First Hospital of Hebei Medical University, Shijiazhuang, Hebei, China; 6 Department of Traumatic Surgery, The First Hospital of Hebei Medical University, Shijiazhuang, Hebei, China; 7 Department of Social Medicine and Health Services Management, Faculty of Public Health, Hebei Medical University, Shijiazhuang, Hebei, China; 8 Hebei Province Key Laboratory of Environment and Human Health, Shijiazhuang, Hebei, China

**Keywords:** 25-hydroxyvitamin D, dietary vitamin D intake, health examination, indoor lifestyle, northern China, physical activity, sunlight exposure, vitamin D deficiency

## Abstract

**Background/objectives:**

Vitamin D deficiency is not confined to traditionally defined high-risk groups, yet its prevalence and modifiable behavioral correlates among apparently healthy adults remain poorly characterized, particularly in northern China. This study assessed vitamin D status among adults undergoing routine health examinations in Hebei Province and examined whether modifiable behavioral factors were independently associated with deficiency risk.

**Methods:**

A cross-sectional study was conducted among 4,811 adults attending routine health examinations in Hebei Province, China, after exclusion of participants with documented major chronic conditions known to affect vitamin D metabolism. Serum 25-hydroxyvitamin D [25(OH)D] was measured, and vitamin D deficiency was defined as <20 ng/mL. Structured questionnaires were used to collect information on sunlight exposure, physical activity, dietary vitamin D intake, and vitamin D-containing supplement use. Multivariable logistic regression models were applied to examine associations between these behavioral factors and vitamin D deficiency, with adjustment for sex, age group, smoking status, alcohol consumption, BMI category, and season of blood collection. A cumulative behavioral score was constructed to assess the combined association of favorable behavioral factors with deficiency risk.

**Results:**

The median serum 25(OH)D concentration was 20.5 ng/mL (IQR 15.4–26.6), and 47.5% of participants were vitamin D deficient, while only 15.3% had sufficient vitamin D status. Deficiency was more prevalent in women, younger and working-age adults, and participants examined during winter. In the fully adjusted model, sunlight exposure ≥30 min/day (OR 0.677, 95% CI 0.599–0.765), moderate-to-high physical activity (OR 0.773, 95% CI 0.678–0.880), adequate dietary vitamin D intake (OR 0.734, 95% CI 0.651–0.828), and vitamin D supplementation (OR 0.797, 95% CI 0.705–0.902) were each associated with lower odds of deficiency. A graded inverse association was observed for the cumulative behavioral score; compared with participants with no favorable behavioral factors, those with four had substantially lower odds of deficiency (OR 0.285, 95% CI 0.189–0.423).

**Conclusion:**

Vitamin D deficiency was highly prevalent among adults attending routine health examinations in northern China after exclusion of major chronic conditions known to affect vitamin D metabolism. The burden was concentrated in women, younger and working-age adults, and winter months, while lower deficiency risk was consistently associated with more favorable behavioral patterns. The observed distribution is consistent with an indoor-lifestyle framework in which vitamin D status is shaped not only by the environmental availability of sunlight, but also by the extent to which daily routines permit effective ultraviolet exposure.

## 1 Introduction

Vitamin D deficiency is commonly framed as a problem of clinically vulnerable populations. Yet an equally important question is why it remains widespread among adults who appear healthy and live in environments where sunlight is seasonally available. Vitamin D is a fat-soluble prohormone with well-established roles in calcium and phosphorus homeostasis, skeletal integrity, immune regulation, and metabolic function (1, 2). Beyond its classical role in bone health, suboptimal vitamin D status has been linked to a broad range of adverse health outcomes, including immune dysregulation and metabolic disorders (1–4). For most populations, cutaneous synthesis driven by solar ultraviolet B radiation represents the principal route of vitamin D acquisition, making sunlight exposure a central determinant of population-level status (5, 6).

The environmental availability of sunlight does not necessarily translate into adequate vitamin D status. Populations living in settings with sufficient ambient sunlight might be expected to have relatively low rates of deficiency. Epidemiological evidence, however, consistently challenges this assumption. High rates of vitamin D insufficiency and deficiency have been reported even in sun-rich or relatively low-latitude settings, indicating that vitamin D status is shaped not only by environmental availability but also by the extent to which daily life permits that availability to be realized (7–14). In contemporary populations, the central question may therefore be not whether sunlight is environmentally available, but whether daily routines allow it to become effective physiological exposure—a distinction particularly salient in populations whose routines are increasingly confined to indoor settings.

In China, inadequate vitamin D status has been documented in several population groups, including children, older adults, and individuals with chronic diseases (15–20). Evidence among apparently healthy adults, however, remains comparatively limited. Although recent studies in adult health examination populations have begun to characterize vitamin D status across demographic and seasonal dimensions (21, 22), they provide limited insight into why deficiency remains common in non-clinical adult populations. Whether modifiable behavioral factors are independently associated with deficiency risk in this population has not been well characterized.

Hebei Province provides a useful setting in which to examine this issue. Located in northern China, Hebei experiences marked seasonal variation in solar radiation and daylight duration across the year (23, 24). At the same time, many adults in this setting may have limited effective sunlight exposure because of indoor-dominant work and living patterns, reduced outdoor activity, and potentially insufficient intake of vitamin D-rich foods (6, 12–14). In this context, ambient ultraviolet availability alone cannot be assumed to confer adequate physiological exposure. Assessing vitamin D status in Hebei therefore offers an opportunity to examine whether deficiency persists in an apparently healthy adult population and whether its distribution reflects limited behavioral access to sunlight.

The present study was designed to address this question among apparently healthy adults undergoing routine health examinations in Hebei Province. Specifically, it aimed to assess the prevalence of vitamin D deficiency, examine whether modifiable behavioral factors were independently associated with deficiency risk, and interpret the observed pattern within an indoor-lifestyle framework.

## 2 Materials and methods

### 2.1 Study design and participants

This cross-sectional study was conducted among adults undergoing routine health examinations at the Health Management Center of the First Hospital of Hebei Medical University between December 2024 and December 2025. Adults aged ≥18 years who had resided in Hebei Province for at least 1 year and had available serum 25-hydroxyvitamin D [25(OH)D] measurements were eligible for inclusion. Participants were classified as apparently healthy on the basis of documented medical history and available clinical records obtained during the health examination process. This classification referred specifically to the absence of major chronic conditions known to affect vitamin D metabolism, was based on available examination records rather than exhaustive diagnostic screening, and should not be interpreted as indicating absence of cardiometabolic risk factors. Participants with chronic liver disease, chronic kidney disease, gastrointestinal resection, organ transplantation, malignancy, diabetes mellitus, or use of medications affecting vitamin D metabolism, such as glucocorticoids, antiepileptic drugs, and rifampicin, were excluded. Overweight/obesity and smoking were not exclusion criteria and were included as covariates in the multivariable models.

**Figure 1:**
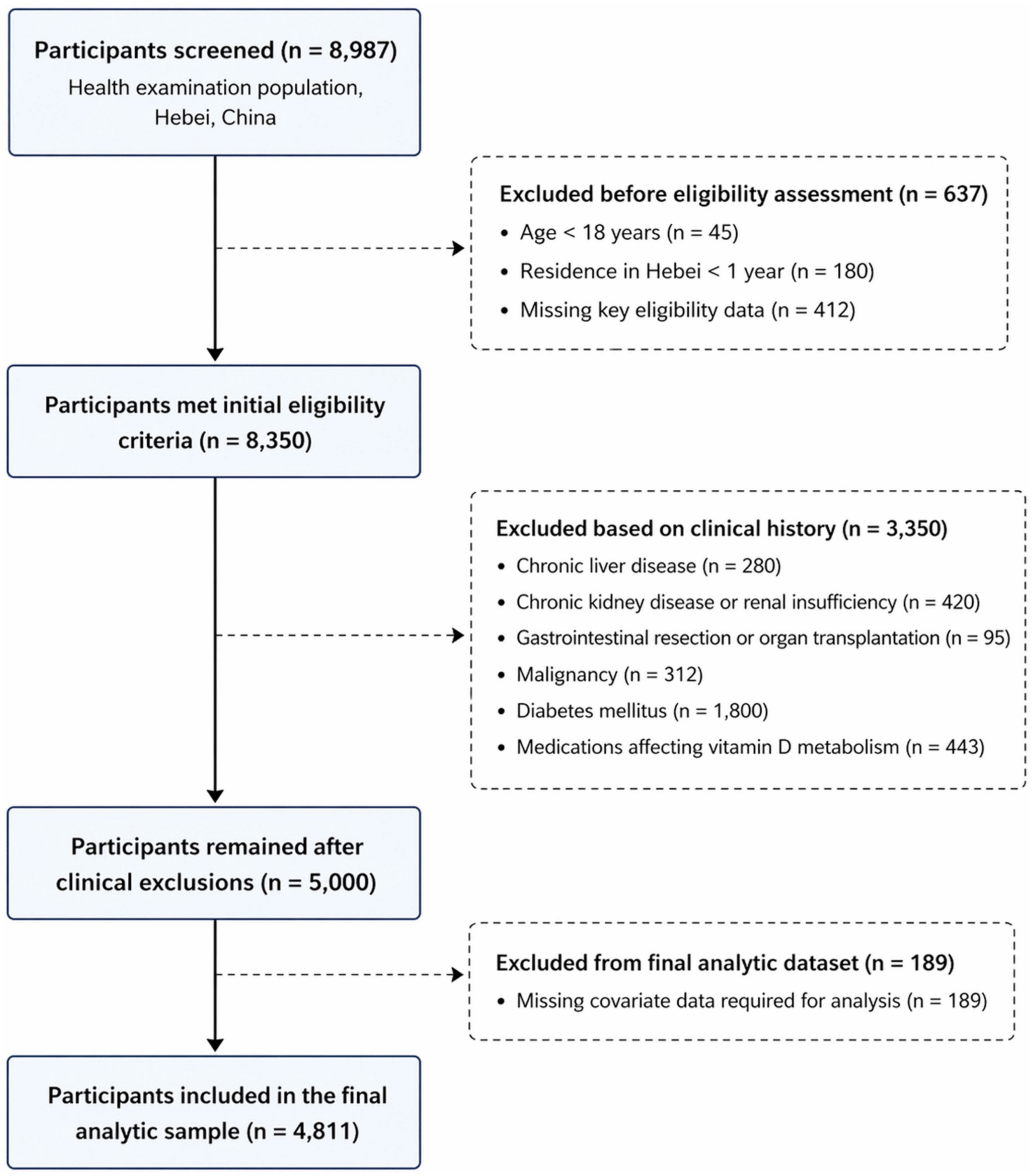
Flowchart of participant selection. A total of 8,987 individuals undergoing routine health examinations were screened. After application of eligibility and clinical exclusion criteria, 5,000 participants remained eligible. Of these, 189 were excluded because of missing data in variables required for the analytic dataset, leaving 4,811 participants in the final analytic sample. Participants meeting multiple exclusion criteria were categorized according to the primary recorded reason.

Participants meeting multiple exclusion criteria were assigned to the primary recorded reason for exclusion. Participants were further categorized by sex, age group (18–29, 30–59, and ≥60 years), and season of blood collection. Season was classified according to calendar month as spring (March–May), summer (June–August), autumn (September–November), and winter (December–February). The participant selection process is shown in Figure 1.

### 2.2 Laboratory measurements

Fasting venous blood samples were collected in the morning after an overnight fast of at least 8 h. Serum 25(OH)D testing was performed as part of the routine clinical laboratory workflow of the health examination center. Blood collection, specimen handling, and biochemical testing followed the standard operating procedures of the hospital clinical laboratory. As this was a retrospective analysis of routinely collected health examination data, detailed research-specific pre-analytical parameters, such as centrifugation time, storage temperature, and duration of sample preservation before analysis, were not separately recorded. Serum 25(OH)D concentrations were measured using electrochemiluminescence immunoassay on a Roche Cobas e601 analyzer (Roche Diagnostics, Mannheim, Germany), with intra- and inter-assay coefficients of variation <10%. Vitamin D status was categorized using commonly applied cut-points in previous clinical and epidemiological studies: deficiency, <20 ng/mL; insufficiency, 20–29 ng/mL; and sufficiency, ≥30 ng/mL (25, 26). These categories were used as epidemiological definitions for analytical comparability with prior studies and should not be interpreted as a recommendation for universal screening or treatment in asymptomatic adults. Additional laboratory markers, including hematologic, hepatic, and renal indices, were obtained from routine examination records for exploratory analyses.

### 2.3 Assessment of behavioral factors

Behavioral exposures were assessed using study-specific questionnaires administered at the time of the health examination. The questionnaire items were developed through iterative review by the research team and assessed for face validity and feasibility by clinical and public health researchers prior to implementation. The instrument was designed as a pragmatic behavioral indicator tool for the routine health examination setting rather than a formally validated quantitative exposure assessment scale.

Sunlight exposure was assessed for the preceding month, including average daily outdoor time, timing of outdoor exposure, exposed body parts, sunscreen use, and use of sunshade tools or protective clothing. For the primary analysis, sunlight exposure was dichotomized as <30 min/day or ≥30 min/day according to average daily outdoor time.

Physical activity was assessed using self-reported frequency and typical duration of moderate-to-vigorous activities, including brisk walking, running, cycling, and fitness training. Participants reporting a typical session duration of ≥60 min were classified as having moderate-to-high physical activity, whereas all others, including those reporting no activity, were classified as having low physical activity. This classification was based on typical session duration and was used as a study-specific indicator of higher habitual activity rather than adherence to guideline-recommended activity levels (27).

Dietary vitamin D intake was assessed using a brief food-frequency questionnaire covering seven vitamin D-containing food groups, including fatty fish, egg yolk, animal liver, sun-exposed mushrooms, and vitamin D-fortified foods. Each item was scored from 0 to 3 according to consumption frequency, yielding a total dietary vitamin D intake score ranging from 0 to 21. In the absence of a validated Chinese dietary vitamin D scoring threshold for this population, the classification threshold was defined *a priori* as the sample median. Participants scoring above the median were classified as having adequate dietary vitamin D intake, whereas those scoring at or below the median were classified as having low intake.

Vitamin D supplementation during the preceding 3 months was analyzed as a binary variable and included any self-reported use of vitamin D-containing preparations, including vitamin D-only supplements, multivitamins, or combination preparations such as calcium–vitamin D products. Participants reporting occasional or regular use were classified as supplement users, whereas those reporting no use were classified as non-users. Information on supplement type, dose, frequency, duration, and adherence was not standardized.

Smoking status was recorded as never, former, or current smoker. For multivariable analyses, current smokers were classified as smokers, and never or former smokers were classified as non-smokers. Alcohol consumption was recorded and analyzed as a binary variable. A cumulative favorable behavioral score was constructed by summing four favorable behaviors: sunlight exposure ≥30 min/day, moderate-to-high physical activity, adequate dietary vitamin D intake, and vitamin D supplementation. The score ranged from 0 to 4, with higher values indicating a greater number of favorable behavioral factors.

### 2.4 Ultraviolet exposure data

Monthly mean ultraviolet (UV) exposure data were obtained from the NASA Prediction Of Worldwide Energy Resources (POWER) Data Access Viewer for Shijiazhuang, China. Monthly mean UV exposure was assigned to each participant according to the month of blood collection and used as a proxy for environmental exposure. Because individual residential coordinates were not available, city-level monthly UV exposure was used rather than individual-level ambient exposure. Restricted cubic spline models were applied to examine the potential nonlinear association between monthly mean UV exposure and vitamin D deficiency as an exploratory analysis. Although UV exposure was assigned based on monthly values, season of blood collection was used in the primary regression models to account for broader seasonal variation and to improve model interpretability. This analysis was exploratory and was not intended to identify clinical thresholds.

### 2.5 Physical examination

Height and weight were measured using standardized protocols. Body mass index (BMI) was calculated as weight in kilograms divided by height in meters squared and categorized according to Chinese adult criteria: underweight, <18.5 kg/m^2^; normal weight, 18.5–23.9 kg/m^2^; overweight, 24.0–27.9 kg/m^2^; and obesity, ≥28.0 kg/m^2^ (28). BMI category was included as a prespecified anthropometric covariate in multivariable analyses.

### 2.6 Statistical analysis

All statistical analyses were performed using R software (version 4.5.2). Serum 25(OH)D concentration was analyzed both as a continuous variable and as a categorical outcome. Vitamin D deficiency was defined as serum 25(OH)D < 20 ng/mL using the predefined epidemiological cut-point described in Section 2.2. Categorical variables were presented as counts and percentages and compared using the χ^2^ test. Continuous variables were expressed as medians with interquartile ranges (IQRs) and compared using the Wilcoxon rank-sum test or Kruskal–Wallis test, as appropriate.

Multivariable logistic regression was used to estimate the associations between behavioral factors and vitamin D deficiency, with odds ratios (ORs) and 95% confidence intervals (CIs) reported. Three sequential models were fitted. Model 1 was adjusted for sex and age group. Model 2 was further adjusted for smoking status, alcohol consumption, and BMI category. Model 3 was additionally adjusted for season of blood collection. Physical activity, sunlight exposure, dietary vitamin D intake, and vitamin D supplementation were first examined as individual behavioral factors. The cumulative favorable behavioral score, defined as the number of favorable behaviors present and ranging from 0 to 4, was analyzed in a separate model to avoid collinearity with the individual behavioral factors.

Restricted cubic spline analyses with three knots were conducted as exploratory analyses to assess potential nonlinear associations between monthly mean UV exposure and vitamin D deficiency, and between serum 25(OH)D concentration and selected routine laboratory markers (29). Spline models were adjusted for sex, age group, BMI category, smoking status, alcohol consumption, and season of blood collection. *p* values for overall, linear, and nonlinear associations were reported. No correction for multiple comparisons was applied to these exploratory analyses. A two-sided *p* value <0.05 was considered statistically significant.

## 3 Results

### 3.1 Participant characteristics and overall vitamin D status

A total of 4,811 apparently healthy adults were included in the analysis. Vitamin D deficiency was common in this population, affecting 2,285 participants (47.5%), while 1,790 (37.2%) were classified as insufficient and only 736 (15.3%) had sufficient vitamin D status. The median serum 25(OH)D concentration was 20.5 ng/mL (IQR 15.4–26.6). Participants were predominantly female (54.5%), with the largest proportion aged 30–59 years (54.6%). Most individuals were classified as overweight or obese (61.6%). Behavioral characteristics indicated that more than half of participants reported less than 30 min/day of sunlight exposure (56.7%) and low physical activity (68.7%); 57.0% had low dietary vitamin D intake, and 60.6% reported occasional or regular vitamin D supplementation during the preceding 3 months. Participant characteristics and the distribution of vitamin D status are presented in Table 1.

**Table 1:** Serum 25(OH)D concentrations and vitamin D status according to participant characteristics.

Variable	n (%)	25(OH)D, ng/mL, Median (IQR)	Deficiency n (%)	Insufficiency n (%)	Sufficiency n (%)	*p* value
Overall	4,811 (100)	20.5 (15.4, 26.6)	2,285 (47.5)	1790 (37.2)	736 (15.3)	—
Sex						<0.001
Male	2,187 (45.5)	21.6 (16.3, 26.6)	894 (40.9)	982 (44.9)	311 (14.2)	
Female	2,624 (54.5)	19.2 (14.7, 26.5)	1,391 (53.0)	808 (30.8)	425 (16.2)	
Age group						<0.001
18–29 years	891 (18.5)	17.0 (13.9, 22.9)	544 (61.1)	248 (27.9)	99 (11.0)	
30–59 years	2,629 (54.6)	20.5 (15.8, 26.7)	1,245 (47.4)	986 (37.5)	398 (15.1)	
≥60 years	1,291(26.8)	21.9 (16.4, 27.8)	496 (38.4)	556 (43.1)	239 (18.5)	
BMI category						<0.001
Underweight (<18.5 kg/m^2^)	141 (2.9)	15.9 (14.2, 21.4)	96 (68.1)	34 (24.1)	11 (7.8)	
Normal weight (18.5–23.9)	1708 (35.5)	20.4 (15.1, 27.4)	809 (47.4)	585 (34.3)	314 (18.4)	
Overweight (24.0–27.9)	1929 (40.1)	21.0 (15.7, 26.9)	865 (44.8)	759 (39.3)	305 (15.8)	
Obese (≥28.0)	1,033 (21.5)	20.1 (15.4, 25.5)	515 (49.9)	412 (39.9)	106 (10.3)	
Smoking status						0.015
Yes	864 (18.0)	21.5 (16.3, 26.5)	355 (41.1)	401 (46.4)	108 (12.5)	
No	3,947 (82.0)	20.2 (15.2, 26.6)	1930 (48.9)	1,389 (35.2)	628 (15.9)	
Alcohol consumption						0.468
Yes	1,338 (27.8)	21.4 (15.6, 26.0)	590 (44.1)	594 (44.4)	154 (11.5)	
No	3,473 (72.2)	20.2 (15.1, 26.8)	1,695 (48.8)	1,196 (34.4)	582 (16.8)	
Sunlight exposure						<0.001
<30 min/day	2,729 (56.7)	19.2 (14.6, 25.5)	1,445 (52.9)	956 (35.0)	328 (12.0)	
≥30 min/day	2082 (43.3)	21.6 (16.3, 28.0)	840 (40.3)	834 (40.1)	408 (19.6)	
Physical activity						<0.001
Low	3,303 (68.7)	20.0 (15.0, 26.0)	1,656 (50.1)	1,191 (36.1)	456 (13.8)	
Moderate-to-high (≥60 min/session)	1,508 (31.3)	21.3 (15.9, 27.6)	629 (41.7)	599 (39.7)	280 (18.6)	
Dietary vitamin D intake						0.003
Low	2,743 (57.0)	19.9 (15.0, 26.2)	1,390 (50.7)	970 (35.4)	383 (14.0)	
Adequate	2068 (43.0)	21.3 (15.9, 27.2)	895 (43.3)	820 (39.7)	353 (17.1)	
Vitamin D supplementation						0.001
Yes, occasional or regular use	2,917 (60.6)	21.2 (15.8, 26.6)	1,451 (49.7)	1,016 (34.8)	450 (15.4)	
No	1894 (39.4)	20.1 (15.0, 26.5)	834 (44.0)	774 (40.9)	286 (15.1)	
Season of blood collection						<0.001
Spring (March–May)	1,227 (25.5)	20.2 (15.0, 25.0)	605 (49.3)	472 (38.5)	150 (12.2)	
Summer (June–August)	1,018 (21.2)	21.4 (16.7, 27.4)	406 (39.9)	425 (41.7)	187 (18.4)	
Autumn (September–November)	1,657 (34.4)	22.0 (16.2, 28.4)	707 (42.7)	657 (39.6)	293 (17.7)	
Winter (December–February)	909 (18.9)	17.0 (13.8, 22.7)	568 (62.5)	236 (26.0)	105 (11.6)	

Values are presented as n (%), median (IQR), or both, as appropriate. Vitamin D deficiency was defined as serum 25(OH)D < 20 ng/mL, insufficiency as 20–29 ng/mL, and sufficiency as ≥30 ng/mL. In this table, vitamin D supplementation refers to any self-reported occasional or regular use of vitamin D-containing preparations, including vitamin D-only supplements, multivitamins, or combination preparations.

### 3.2 Distribution of vitamin D status across sex, age, and season

**Figure 2:**
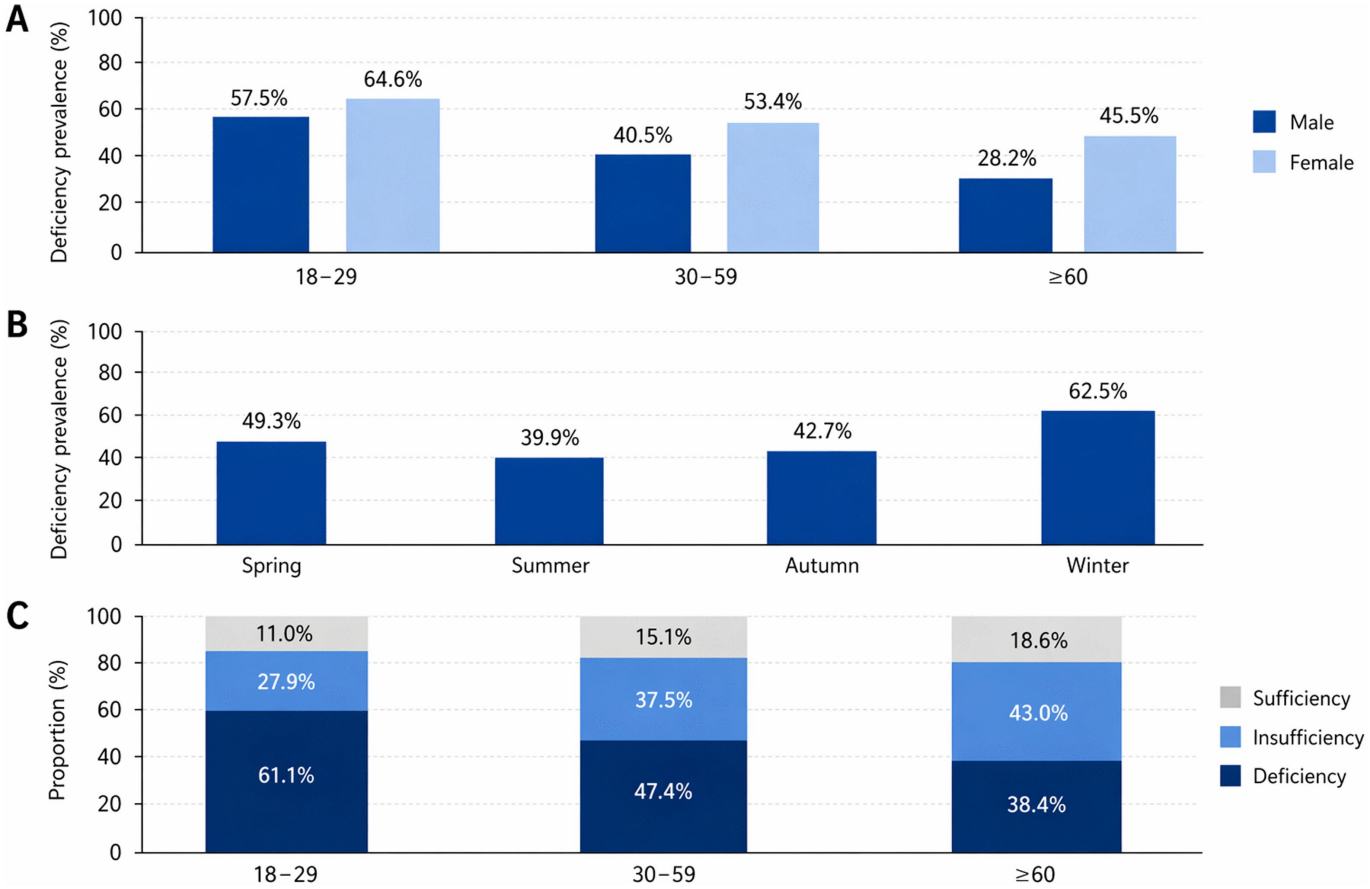
Distribution of vitamin D deficiency and vitamin D status by age group, sex, and season. **(A)** Prevalence of vitamin D deficiency by age group and sex; **(B)** seasonal prevalence of vitamin D deficiency; **(C)** distribution of vitamin D deficiency, insufficiency, and sufficiency across age groups. Vitamin D deficiency was defined as serum 25(OH)D < 20 ng/mL, insufficiency as 20–29 ng/mL, and sufficiency as ≥30 ng/mL. Values are presented as percentages.

Vitamin D deficiency showed clear differences across sex, age groups, and season of blood collection. Women had a higher prevalence of deficiency than men (53.0% vs. 40.9%) and a lower median serum 25(OH)D concentration [19.2 ng/mL (IQR 14.7–26.5) vs. 21.6 ng/mL (IQR 16.3–26.6)]. Across age groups, deficiency was most prevalent among participants aged 18–29 years and least prevalent among those aged ≥60 years, decreasing from 61.1 to 47.4 and 38.4%, respectively. Marked seasonal variation was also observed. Vitamin D deficiency was most prevalent in winter (62.5%), followed by spring (49.3%), autumn (42.7%), and summer (39.9%). Median serum 25(OH)D concentrations showed a corresponding seasonal pattern, with the lowest level observed in winter [17.0 ng/mL (IQR 13.8–22.7)] and the highest in autumn [22.0 ng/mL (IQR 16.2–28.4)] (Figure 2).

### 3.3 Behavioral and multivariable correlates of vitamin D deficiency

Favorable behavioral factors were consistently associated with lower odds of vitamin D deficiency in multivariable logistic regression models (Table 2). In the fully adjusted model, sunlight exposure ≥30 min/day was associated with lower odds of deficiency compared with <30 min/day (OR 0.677, 95% CI 0.599–0.765). Similar inverse associations were observed for moderate-to-high physical activity (OR 0.773, 95% CI 0.678–0.880), adequate dietary vitamin D intake (OR 0.734, 95% CI 0.651–0.828), and vitamin D supplementation (OR 0.797, 95% CI 0.705–0.902). Vitamin D supplementation showed a higher crude prevalence of deficiency in Table 1, whereas it was associated with lower odds of deficiency after multivariable adjustment in Table 2.

**Table 2:** Associations of behavioral factors with vitamin D deficiency.

Variable	Category	Model 1, OR (95% CI)	Model 2, OR (95% CI)	Model 3, OR (95% CI)
Physical activity	Low (Ref)			
	Moderate-to-high	0.781 (0.687, 0.888)	0.765 (0.672, 0.870)	0.773 (0.678, 0.880)
Sunlight exposure	<30 min/day (Ref)			
	≥30 min/day	0.643 (0.571, 0.725)	0.648 (0.574, 0.731)	0.677 (0.599, 0.765)
Dietary vitamin D intake	Low (Ref)			
	Adequate	0.720 (0.639, 0.810)	0.725 (0.644, 0.817)	0.734 (0.651, 0.828)
Vitamin D supplementation	No (Ref)			
	Yes	0.782 (0.692, 0.883)	0.783 (0.693, 0.885)	0.797 (0.705, 0.902)
Number of favorable behavioral factors	None (Ref)			
	1	0.781 (0.653, 0.933)	0.772 (0.645, 0.923)	0.810 (0.675, 0.970)
	2	0.507 (0.423, 0.607)	0.502 (0.418, 0.601)	0.541 (0.450, 0.650)
	3	0.424 (0.343, 0.523)	0.420 (0.339, 0.519)	0.450 (0.363, 0.558)
	4	0.257 (0.172, 0.380)	0.258 (0.172, 0.380)	0.285 (0.189, 0.423)

The outcome variable was vitamin D deficiency, defined as serum 25(OH)D < 20 ng/mL. Values are odds ratios (ORs) and 95% confidence intervals (CIs). Model 1 was adjusted for sex and age group. Model 2 was additionally adjusted for smoking status, alcohol consumption, and BMI category. Model 3 was additionally adjusted for season of blood collection. The number of favorable behavioral factors was analyzed in a separate model and was not entered simultaneously with the individual behavioral factors.

A graded inverse association was observed across the cumulative favorable behavioral score. Compared with participants with no favorable behavioral factors, those with one, two, three, and four favorable factors had progressively lower odds of vitamin D deficiency, with ORs of 0.810 (95% CI 0.675–0.970), 0.541 (95% CI 0.450–0.650), 0.450 (95% CI 0.363–0.558), and 0.285 (95% CI 0.189–0.423), respectively (Table 2).

The fully adjusted model additionally revealed associations between vitamin D deficiency and several demographic and anthropometric characteristics (Table 3). Female sex was associated with higher odds of deficiency (OR 1.727, 95% CI 1.481–2.012), whereas older age groups showed lower odds compared with participants aged 18–29 years (30–59 years: OR 0.540, 95% CI 0.458–0.635; ≥60 years: OR 0.357, 95% CI 0.296–0.430). Compared with underweight individuals, participants with normal weight, overweight, and obesity had lower odds of deficiency. Alcohol consumption was associated with slightly higher odds of deficiency (OR 1.216, 95% CI 1.028–1.438), whereas current smoking showed an inverse association.

**Table 3:** Selected variables from the fully adjusted multivariable model of vitamin D deficiency.

Variable	Category	OR (95% CI)
Sex	Male (Ref)	
	Female	1.727 (1.481, 2.012)
Age group	18–29 years (Ref)	
	30–59 years	0.540 (0.458, 0.635)
	≥60 years	0.357 (0.296, 0.430)
BMI category	Underweight (Ref)	
	Normal weight	0.477 (0.324, 0.701)
	Overweight	0.525 (0.357, 0.773)
	Obese	0.627 (0.422, 0.933)
Alcohol consumption	No (Ref)	
	Yes	1.216 (1.028, 1.438)
Smoking status	No (Ref)	
	Yes	0.779 (0.650, 0.933)
Physical activity	Low (Ref)	
	Moderate-to-high	0.748 (0.656, 0.852)
Sunlight exposure	<30 min/day (Ref)	
	≥30 min/day	0.685 (0.606, 0.774)
Dietary vitamin D intake	Low (Ref)	
	Adequate	0.743 (0.658, 0.839)

The outcome variable was vitamin D deficiency, defined as serum 25(OH)D < 20 ng/mL. Values are odds ratios (ORs) and 95% confidence intervals (CIs). The table presents selected variables from the fully adjusted model. The model included sex, age group, BMI category, current smoking, current alcohol consumption, physical activity, sunlight exposure, dietary vitamin D intake, vitamin D supplementation, and season of blood collection. Vitamin D supplementation and season of blood collection were included in the model but are not shown in the table.

### 3.4 Nonlinear association between ultraviolet exposure and vitamin D deficiency

**Figure 3:**
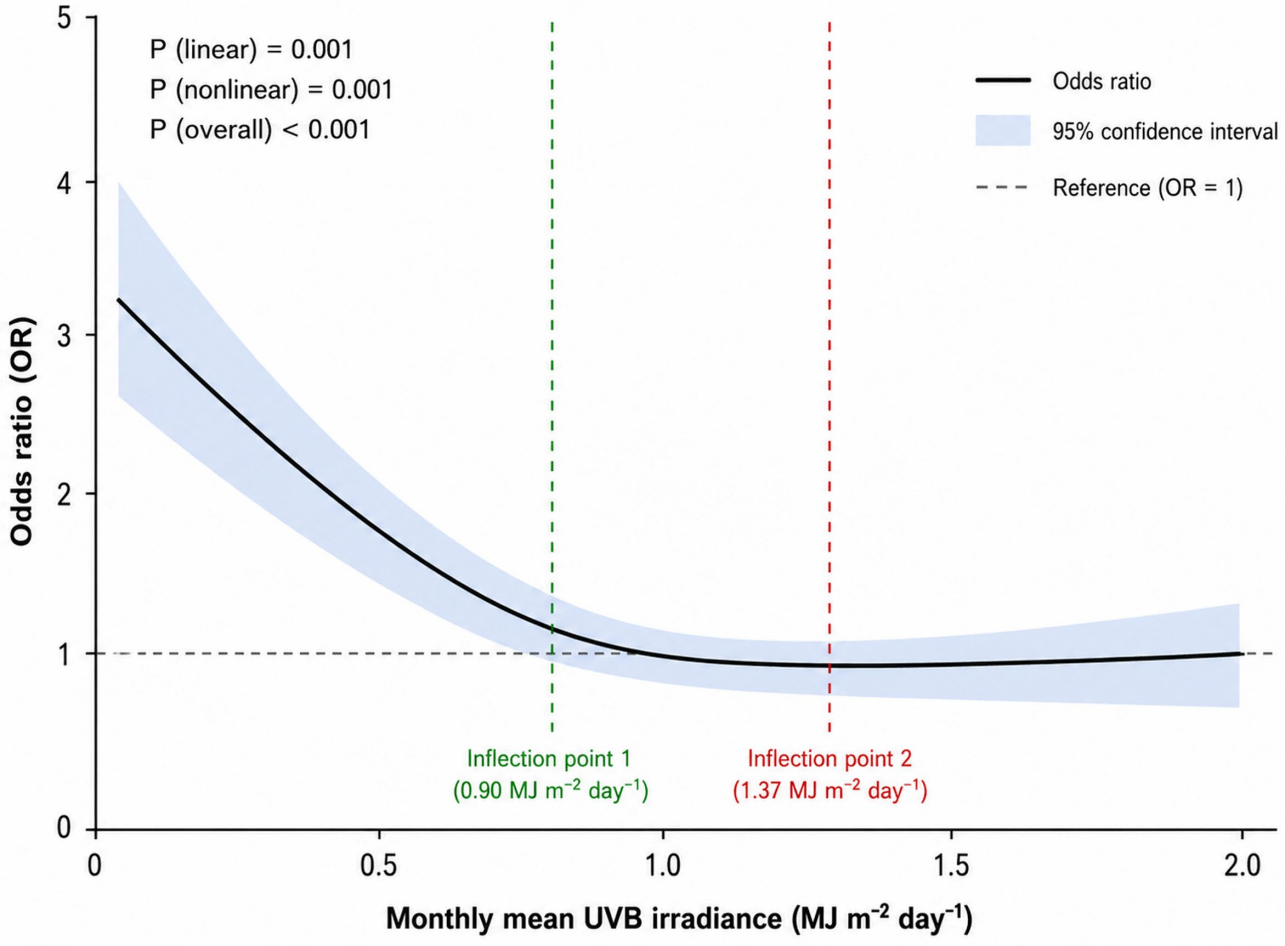
Restricted cubic spline showing the association between ultraviolet (UV) exposure and vitamin D deficiency. The solid line represents the estimated odds ratio, and the shaded area represents the 95% confidence interval. The dashed horizontal line indicates an odds ratio of 1. Vertical dashed lines indicate approximate inflection points from the spline curve and are presented for descriptive purposes only. *p* values for overall association, linearity, and nonlinearity are shown.

Restricted cubic spline analysis was conducted to examine the potential nonlinear association between ultraviolet (UV) exposure and vitamin D deficiency. A significant nonlinear relationship was observed (P for nonlinearity = 0.001; P for overall association < 0.001; Figure 3). The odds of vitamin D deficiency declined steeply with increasing UV exposure at lower levels and then plateaued at higher levels. The curve suggested two inflection points (approximately 0.90 and 1.37), although these should be interpreted descriptively rather than as clinically defined thresholds. As a supplementary exploratory analysis, model discrimination was evaluated using receiver operating characteristic analysis. The fully adjusted model showed an area under the curve of 0.809 for vitamin D deficiency prediction (Supplementary Figure S2). This analysis was used only to describe model discrimination and was not used as the basis for inference regarding behavioral correlates.

## 4 Discussion

Vitamin D deficiency was common among apparently healthy adults undergoing routine health examinations in Hebei Province, yet the main contribution of this study lies in the distribution of that burden rather than in prevalence alone. Deficiency was concentrated among women, younger and working-age adults, and participants examined in winter. Lower odds of deficiency were consistently associated with more favorable behavioral patterns, including longer sunlight exposure, higher physical activity, adequate dietary vitamin D intake, supplementation, and a greater cumulative number of favorable behaviors. This pattern points to a mismatch between environmental sunlight availability and behavioral access to effective ultraviolet exposure.

Among the observed results, the age gradient was the most revealing. Rather than increasing with age, vitamin D deficiency was most prevalent among younger adults and progressively less common in older age groups. This pattern contrasts with the conventional physiological expectation that older adults should be at greater risk because cutaneous vitamin D synthesis declines with age. Similar inverse or attenuated age gradients have been reported in generally healthy populations (30, 31), whereas much of the clinical literature has emphasized older adults because of bone health, frailty, and chronic disease vulnerability (16, 17, 32). The present findings do not contradict the clinical relevance of vitamin D in older populations; instead, they indicate that deficiency in community-dwelling or health examination populations may follow a different distributional logic (21). Younger and working-age adults may be less physiologically vulnerable, but their daily schedules may also provide fewer opportunities for incidental sunlight exposure because of indoor work, fixed routines, and reduced opportunistic outdoor time. Occupational evidence linking indoor work and shift work with lower vitamin D status supports this interpretation (33–36). The age gradient observed here is therefore not an anomaly to be explained away, but a signal that behavioral access to sunlight may be more relevant than biological susceptibility in apparently healthy adult populations. This pattern should not, however, be interpreted as evidence that indoor work routines alone explain vitamin D deficiency among younger adults. Unmeasured factors such as occupation, clothing practices, sunscreen use, commuting patterns, health awareness, previous vitamin D testing, and healthcare-seeking behavior may also have contributed, and the indoor-lifestyle framework should be understood as an interpretive explanation supported by the observed behavioral associations, rather than as a causal mechanism established by this cross-sectional study.

Sex and seasonal differences further clarified this distributional logic. Women had a higher prevalence of deficiency than men, and deficiency peaked in winter, when ultraviolet availability was lowest (6, 22, 23). However, deficiency remained substantial even in summer and autumn, when ambient ultraviolet exposure was relatively higher. Prior evidence from Chinese and other urban populations has linked lower vitamin D status in women to lower outdoor activity, clothing practices, sun avoidance, and other constraints on effective sunlight exposure (15, 16, 18, 19, 37, 38). Sex and season therefore do not represent separate explanations so much as converging indicators of the same underlying issue: environmental ultraviolet availability cannot ensure adequate vitamin D status when daily routines limit biological exposure (7–14).

Behavioral exposures provided the clearest empirical bridge between the observed distribution and the indoor-lifestyle framework. Across progressively adjusted models, sunlight exposure of at least 30 min/day, moderate-to-high physical activity, adequate dietary vitamin D intake, and vitamin D supplementation were each associated with lower odds of deficiency. The cumulative favorable behavioral score showed a graded inverse association across the full range from zero to four favorable behaviors. This gradient is important because it moves the interpretation beyond any single exposure. Sunlight exposure represents the most direct pathway, physical activity may partly increase outdoor time, dietary intake may partly offset limited cutaneous synthesis, and supplementation may partly substitute for constrained cutaneous synthesis while also clustering with other health-maintaining behaviors. The cumulative pattern suggests that vitamin D status is shaped by combinations of everyday behaviors rather than by one isolated factor. A purely latitude-based or season-based explanation cannot fully account for such a gradient. The findings are more consistent with an indoor-lifestyle framework in which sunlight may be environmentally available while remaining behaviorally inaccessible in daily life.

Vitamin D supplementation should be interpreted differently from the other behavioral factors. In the crude distribution, supplement users had a higher prevalence of vitamin D deficiency than non-users, whereas the adjusted model showed lower odds of deficiency among supplement users. This reversal suggests that the crude association was likely influenced by participant characteristics related both to supplement use and deficiency risk, including sex, age, season of blood collection, and health awareness. Although supplementation was associated with lower odds of deficiency after adjustment, this association should not be interpreted as evidence of causal effectiveness in an observational dataset. Reverse causation remains plausible because participants who knew or suspected low vitamin D status may have been more likely to initiate supplementation. The available data also lacked standardized information on dose, frequency, duration, and adherence, all of which are essential for evaluating biological effectiveness. Controlled supplementation studies can estimate efficacy under defined dosing conditions (39), whereas observational data from routine settings often reflect mixed patterns of prior diagnosis, preventive intent, inconsistent use, and incomplete correction of deficiency (40, 41). The present result should therefore be read as a real-world behavioral association rather than an intervention effect.

The spline analysis of ultraviolet exposure added a further layer to the interpretation. The odds of deficiency declined steeply with increasing UV exposure at lower levels and approached a plateau at higher levels, suggesting that the largest marginal reduction in risk occurs when exposure shifts from low to moderate levels (24). Additional increases beyond that range appeared to confer limited incremental benefit. Because this analysis was exploratory, the apparent inflection points should be interpreted descriptively rather than as clinically defined thresholds. Even so, the pattern is compatible with the view that adequate, rather than maximal, sunlight exposure is most relevant for deficiency prevention. It also reinforces the central interpretation that effective access to moderate ultraviolet exposure may matter more than absolute ambient availability.

Not all adjusted associations should be read as mechanistic signals. The lower odds of deficiency among normal-weight, overweight, and obese participants compared with the underweight group do not fit neatly with the common expectation that higher adiposity is associated with lower circulating vitamin D, and may partly reflect the small size and heterogeneity of the underweight subgroup (*n* = 141), within which estimates are likely to be imprecise. The choice of the underweight group as the reference category was made to present odds ratios for all BMI categories relative to the lowest weight stratum; the small size of this subgroup limits the precision of these comparisons and the interpretation of the resulting gradients. The inverse association with current smoking should not be interpreted as protective; residual confounding by occupational routines or greater incidental outdoor exposure among smokers is more plausible, consistent with the occupational evidence linking outdoor work with higher vitamin D status (33–36). The modest positive association with alcohol consumption may similarly reflect broader lifestyle clustering rather than a specific biological pathway. The exploratory spline analyses of laboratory markers are best regarded as hypothesis-generating, given the absence of prespecified hypotheses and the lack of correction for multiple testing.

For prevention, the central message is one of alignment rather than expansion. The findings do not imply that apparently healthy adults should undergo routine biochemical screening, nor do they support indiscriminate population-wide testing. The 25(OH)D thresholds used in this study served as an analytical framework, and current guidance does not support universal screening in generally healthy adults without specific indications (26). The clinical interpretation of vitamin D thresholds in asymptomatic adults remains debated. The <20 ng/mL cut-point used in this study was selected for epidemiological comparability, but alternative thresholds, such as <12 ng/mL for more severe deficiency or <30 ng/mL for insufficiency/low vitamin D status, would change the estimated prevalence and may influence the magnitude and statistical significance of observed associations. Future studies should examine whether the demographic and behavioral patterns reported here remain consistent across different clinically and epidemiologically relevant thresholds. Preventive attention should instead be aligned with how deficiency is distributed in practice. The concentration of deficiency among younger and working-age adults suggests that strategies focused only on conventionally defined high-risk groups may miss an important share of the burden in non-clinical populations. A more proportionate response would emphasize exposure-oriented prevention: recognition of limited effective sunlight exposure in indoor-dominant routines, encouragement of feasible outdoor activity, attention to dietary vitamin D intake, and targeted preventive counseling for adults whose daily schedules leave limited time for outdoor exposure.

The findings should be interpreted in light of several limitations. The cross-sectional design precludes causal inference and does not establish temporal ordering between behaviors and vitamin D status. Behavioral measures were self-reported and based on pragmatic, study-specific instruments designed for a real-world health examination setting rather than standardized surveillance tools. Misclassification of sunlight exposure, physical activity, and dietary vitamin D intake is therefore possible. Although non-differential misclassification may have attenuated true associations, differential reporting related to demographic characteristics, health awareness, previous vitamin D testing, or supplement use cannot be excluded. The dietary assessment was frequency-based rather than quantitatively validated, and supplementation data lacked detailed information on dose and adherence. Apparent health status was determined from documented history and available clinical records rather than exhaustive diagnostic screening, so some subclinical conditions may have remained undetected. Health examination attendees may represent a more health-conscious subset of the broader adult population, potentially leading to underestimation of true community-level deficiency burden. At the same time, the metabolic and behavioral profile of this sample may limit direct transferability to populations with different health-seeking patterns, occupational structures, or activity levels. Residual confounding cannot be excluded, particularly for occupational schedules, clothing practices, sunscreen use, and other determinants of effective ultraviolet exposure. These limitations temper causal interpretation and should be considered when interpreting the observed pattern linking age distribution, behavioral exposures, and the cumulative behavioral gradient in this cohort.

## 5 Conclusion

Vitamin D deficiency was common among adults attending routine health examinations in northern China after exclusion of major chronic conditions known to affect vitamin D metabolism, but the more informative finding was its distribution. Deficiency was concentrated among women, younger and working-age adults, and winter months, and was consistently less frequent among participants with more favorable behavioral patterns. This pattern is consistent with a mismatch between environmental sunlight availability and behavioral access to effective ultraviolet exposure. In this population, vitamin D deficiency appears to reflect not only biological or seasonal risk, but also the way daily routines enable or constrain the conversion of available sunlight into physiological exposure. Future preventive strategies should therefore focus less on expanding indiscriminate screening and more on identifying everyday conditions that limit effective sunlight exposure, outdoor activity, and dietary vitamin D intake.

## Data Availability

The raw data supporting the conclusions of this article will be made available by the authors, without undue reservation.
