# Supplementary material for: Vitamin D deficiency among apparently healthy adults in northern China: behavioral correlates and an indoor-lifestyle framework
Source: Front Public Health. 2026 Jul 8;14:1871195. doi: 10.3389/fpubh.2026.1871195 (PMC13388901; doi:10.3389/fpubh.2026.1871195)
Supplement: Supplementary file 1 [file Data_Sheet_1.ZIP › Supplementary_File2.docx]

**Supplementary File 2. Secondary Exploratory Analyses**

This supplementary file presents secondary exploratory analyses conducted to complement the primary regression analyses. These include restricted cubic spline analyses of associations between serum 25-hydroxyvitamin D [25(OH)D] concentrations and selected routine laboratory markers, and a supplementary assessment of model discrimination for vitamin D deficiency prediction. These analyses were exploratory and should be interpreted as hypothesis-generating rather than confirmatory. No correction for multiple comparisons was applied.

**Supplementary Table S1.** Exploratory restricted cubic spline analyses of associations between serum 25(OH)D concentrations and selected routine laboratory markers

| **Laboratory parameter** | **P for overall** | **P for linear** | **P for nonlinear** |
| --- | --- | --- | --- |
| WBC (×10⁹/L) | <0.001 | <0.001 | <0.001 |
| Hb (g/L) | <0.001 | 0.006 | <0.001 |
| PLT (×10⁹/L) | <0.001 | 0.146 | <0.001 |
| AST (U/L) | <0.001 | 0.066 | 0.011 |
| ALT (U/L) | <0.001 | 0.405 | 0.052 |
| TBIL (μmol/L) | <0.001 | 0.009 | <0.001 |
| BUN (mmol/L) | <0.001 | 0.007 | 0.025 |
| Cr (μmol/L) | <0.001 | <0.001 | <0.001 |

*Note. P for overall indicates the overall association between serum 25(OH)D concentration and each laboratory parameter; P for linear indicates the linear component; P for nonlinear indicates deviation from linearity. All spline models were adjusted for sex, age group, BMI category, smoking status, alcohol consumption, and month of blood collection. These analyses were exploratory and should be interpreted as hypothesis-generating rather than confirmatory. Abbreviations: WBC, white blood cell count; Hb, hemoglobin; PLT, platelet count; ALT, alanine aminotransferase; AST, aspartate aminotransferase; TBIL, total bilirubin; BUN, blood urea nitrogen; Cr, creatinine.*

**Supplementary Figure S1.** Exploratory restricted cubic spline curves for associations between serum 25(OH)D concentrations and selected routine laboratory markers


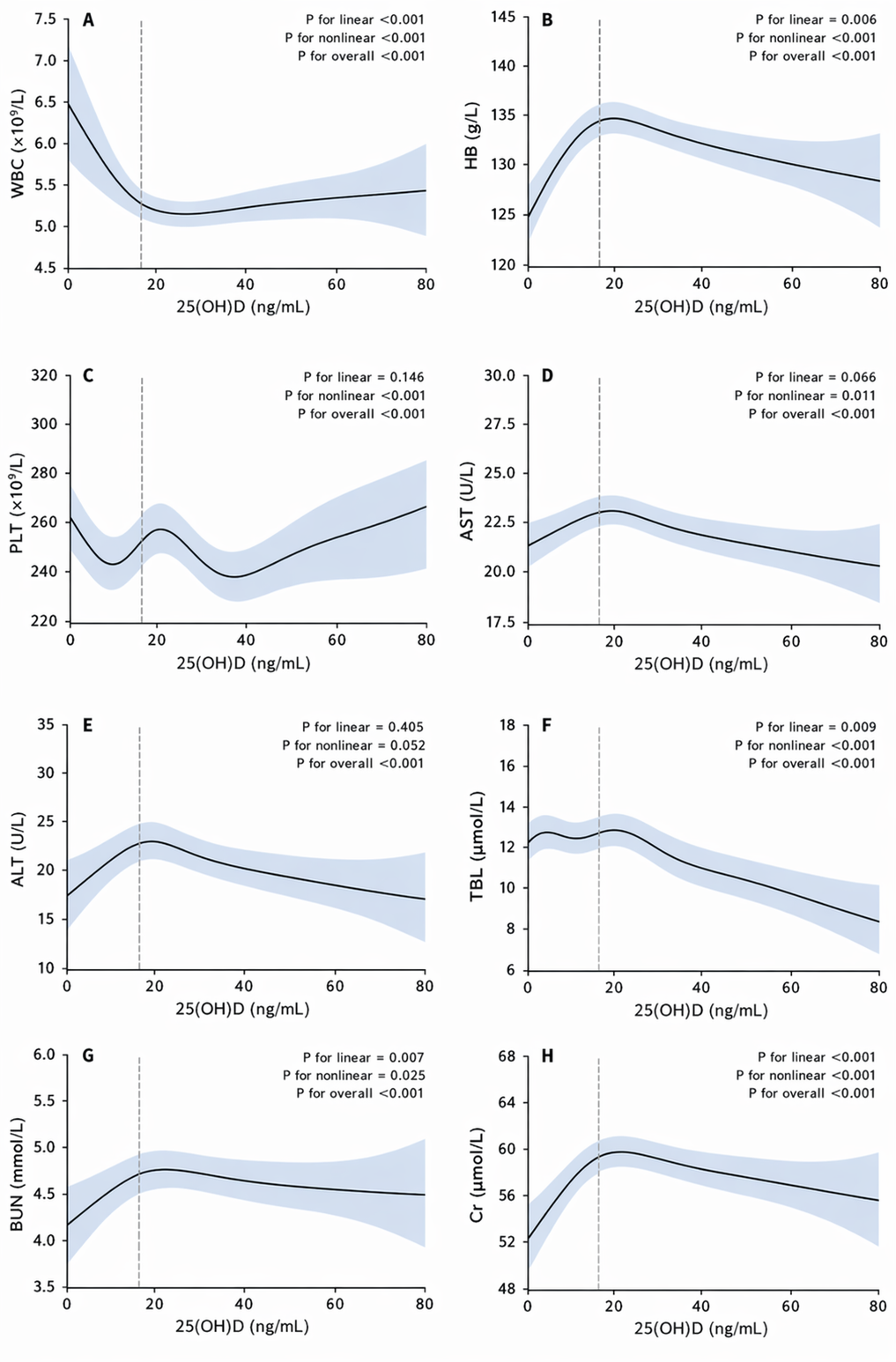


*Note. Panels show the fitted spline curves and corresponding 95% confidence intervals for WBC, Hb, PLT, ALT, AST, TBIL, BUN, and Cr. The dashed vertical line in each panel indicates 25(OH)D = 20 ng/mL, corresponding to the deficiency cut-point used in the primary analysis. All spline models were adjusted for sex, age group, BMI category, smoking status, alcohol consumption, and month of blood collection. These analyses are presented to illustrate potential nonlinear patterns and should not be interpreted as confirmatory evidence of clinical thresholds.*

**Supplementary Figure S2.** Receiver operating characteristic curve for vitamin D deficiency prediction


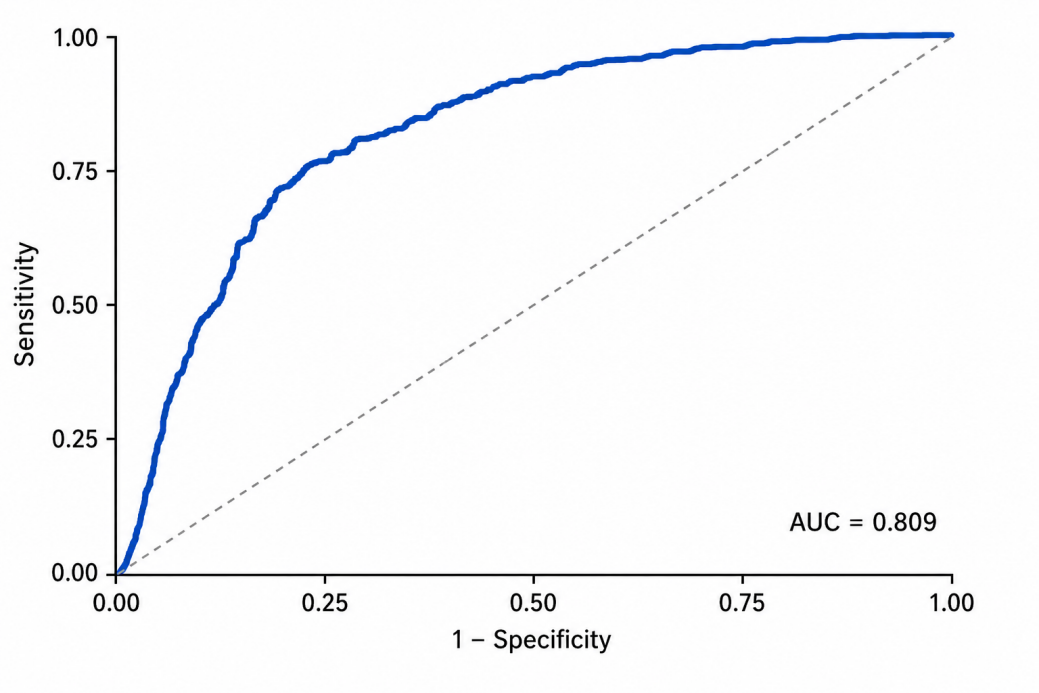


***Note.*** *The fully adjusted logistic regression model showed an area under the receiver operating characteristic curve of 0.809 for vitamin D deficiency prediction. Vitamin D deficiency was defined as serum 25(OH)D <20 ng/mL. This analysis was conducted as a supplementary assessment of model discrimination and was not used as the basis for inference regarding behavioral correlates.*
