# Supplementary material for: Vitamin D deficiency among apparently healthy adults in northern China: behavioral correlates and an indoor-lifestyle framework
Source: Front Public Health. 2026 Jul 8;14:1871195. doi: 10.3389/fpubh.2026.1871195 (PMC13388901; doi:10.3389/fpubh.2026.1871195)
Supplement: Supplementary file 1 [file Data_Sheet_1.ZIP › Supplementary_File1.docx]

**Supplementary File 1. Study-Specific Questionnaires**

This supplementary file presents the study-specific questionnaires used to collect questionnaire-based variables in the present study, including general sociodemographic and lifestyle information (Questionnaire 1), sunlight exposure during the preceding month (Questionnaire 2), and dietary vitamin D intake and supplement use (Questionnaire 3). The derivation of analytic exposure variables from questionnaire responses is described in the Methods section of the main manuscript.

*Note on reference time windows: sunlight exposure items refer to the preceding one month; dietary intake items refer to the preceding one month; supplement use items refer to the preceding three months.*

**Questionnaire 1: General Information and Lifestyle Questionnaire**

**Purpose:** *To collect basic sociodemographic information and lifestyle-related factors that may influence vitamin D status.*

**A. Sociodemographic Information**

1. Sex:

□ Male

□ Female

2. Year of birth: ______

3. Place of residence:

□ Urban

□ Rural

4. Occupation type (in relation to sunlight exposure):

□ Mainly indoor work

□ Mainly outdoor work

□ Mixed indoor and outdoor work

**For female participants only:**

Menopausal status:

□ Premenopausal

□ Postmenopausal

*If postmenopausal, age at menopause: ______ years*

**B. Smoking and Alcohol Consumption**

Smoking status:

□ Never

□ Former

□ Current

*If current smoker — cigarettes per day: ______; duration: ______ years*

Alcohol consumption:

□ Never

□ Occasionally

□ 1–2 days/week

□ 3–5 days/week

□ Almost every day

**C. Physical Activity**

Frequency of moderate-to-vigorous physical activity (e.g., brisk walking, running, cycling, fitness training):

□ 0 times/week

□ 1–2 times/week

□ 3–4 times/week

□ ≥5 times/week

Typical duration per session:

□ <30 min

□ 30–59 min

□ ≥60 min

**D. Supplement Use**

Vitamin D supplementation in the past 3 months:

□ Never

□ Occasionally

□ Regularly

*If yes, estimated dose (if known): ______ IU/day*

Calcium supplementation:

□ Yes

□ No

Multivitamin supplementation:

□ Yes

□ No

**Questionnaire 2: Sunlight Exposure Questionnaire (Past 1 Month)**

**Instruction:** *Please complete this questionnaire according to your usual behaviors during the one month preceding the health examination. For each question, select the option that best represents your typical situation. Responses will be used only for scientific research and kept strictly confidential.*

**A. Daily Outdoor Time**

1. Average daily outdoor time:

□ <15 min/day

□ 15–29 min/day

□ 30–59 min/day

□ ≥60 min/day

**B. Timing of Outdoor Exposure**

2. Usual time of outdoor exposure:

□ Mainly during 10:00–15:00

□ Mainly before 10:00 or after 15:00

□ Uncertain

**C. Clothing Coverage and Skin Exposure**

3. Main body parts typically exposed to sunlight:

□ Face and hands only

□ Face, hands, and forearms

□ Face, hands, forearms, and lower legs

□ Most of the body

**D. Sunscreen Use**

4. Frequency of sunscreen use (SPF ≥30):

□ Never

□ Occasionally (≤2 days/week)

□ Frequently (3–4 days/week)

□ Almost always (≥5 days/week)

**E. Sunshade Tools and Clothing**

5. Frequency of use of sunshade tools or protective clothing:

□ Never

□ Occasionally (≤2 days/week)

□ Frequently (3–4 days/week)

□ Almost always (≥5 days/week)

6. Types of sunshade tools used (select all that apply):

□ Hat or cap

□ Parasol or umbrella

□ Gloves

□ UV-protective clothing

□ Other: ___________

**Questionnaire 3: Dietary Vitamin D Intake Assessment**

**Instruction:** *This questionnaire assesses your usual dietary intake of vitamin D–rich foods over the past month. For each item, please select the option that best describes your average consumption frequency.*

**A. Natural Vitamin D–Rich Foods**

1. Fatty fish (e.g., salmon, tuna, mackerel, sardines):

□ Never/rarely

□ 1–2 times/week

□ 3–4 times/week

□ ≥5 times/week

2. Egg yolk:

□ Never/rarely

□ 1–2 times/week

□ 3–4 times/week

□ ≥5 times/week

3. Animal liver (e.g., pork or chicken liver):

□ Never/rarely

□ 1–2 times/week

□ 3–4 times/week

□ ≥5 times/week

4. Mushrooms exposed to sunlight (e.g., dried mushrooms, shiitake):

□ Never/rarely

□ 1–2 times/week

□ 3–4 times/week

□ ≥5 times/week

**B. Vitamin D–Fortified Foods**

5. Vitamin D–fortified milk or dairy products:

□ Never/rarely

□ 1–2 times/week

□ 3–4 times/week

□ ≥5 times/week

6. Vitamin D–fortified yogurt or beverages:

□ Never/rarely

□ 1–2 times/week

□ 3–4 times/week

□ ≥5 times/week

7. Vitamin D–fortified cereals or grain products:

□ Never/rarely

□ 1–2 times/week

□ 3–4 times/week

□ ≥5 times/week

**C. Supplement Use**

8. Vitamin D supplementation in the past 3 months:

□ Never

□ Occasionally

□ Regularly

*If regularly, approximate daily dose (if known): ______ IU/day*

9. Calcium supplements or multivitamin supplements containing vitamin D:

□ Yes

□ No

**Derivation of the Physical Activity Classification Variable**

Physical activity was classified using both self-reported weekly frequency and typical session duration from Questionnaire 1 (Section C). Total weekly moderate-to-vigorous physical activity was estimated by combining reported frequency category with typical session duration category. Participants with an estimated total activity time of ≥150 min/week were classified as having moderate-to-high physical activity; those with <150 min/week, including those reporting no activity, were classified as having low physical activity. This classification was intended to approximate guideline-recommended activity levels.

Note. Activity types assessed are consistent with moderate-to-vigorous physical activity as described in the WHO 2020 guidelines on physical activity and sedentary behaviour. Because both frequency and duration were self-reported using categorical response options, the derived weekly activity variable should be interpreted as an approximate classification rather than a precise measure of weekly energy expenditure.

**Derivation of the Dietary Vitamin D Intake Score**

Each of the seven dietary items in Questionnaire 3 (items 1–7) was scored on a four-point ordinal scale as follows: never/rarely = 0; 1–2 times/week = 1; 3–4 times/week = 2; ≥5 times/week = 3. Item scores were summed to produce a total dietary vitamin D intake score (range 0–21). Higher scores indicate greater habitual frequency of consumption of vitamin D–containing foods.

Given the absence of a validated Chinese dietary vitamin D scoring instrument with established cut-points applicable to this population, the classification threshold was defined a priori as the sample median total score. Participants scoring above the median were classified as having adequate dietary vitamin D intake; those scoring at or below the median were classified as having low dietary vitamin D intake. This approach was pre-specified before data analysis and is consistent with prior cross-sectional nutritional studies employing study-specific dietary indices in the absence of externally validated thresholds.

*Note. Supplement use items (Questionnaire 3, items 8–9) were not included in the dietary vitamin D intake score. Vitamin D supplementation was analyzed as a separate binary variable (any use vs. no use) in the regression analyses, as described in the Methods section of the main manuscript.*
