# Supplementary figures and images for: Vitamin D deficiency among apparently healthy adults in northern China: behavioral correlates and an indoor-lifestyle framework
Source: Front Public Health. 2026 Jul 8;14:1871195. doi: 10.3389/fpubh.2026.1871195 (PMC13388901; doi:10.3389/fpubh.2026.1871195)

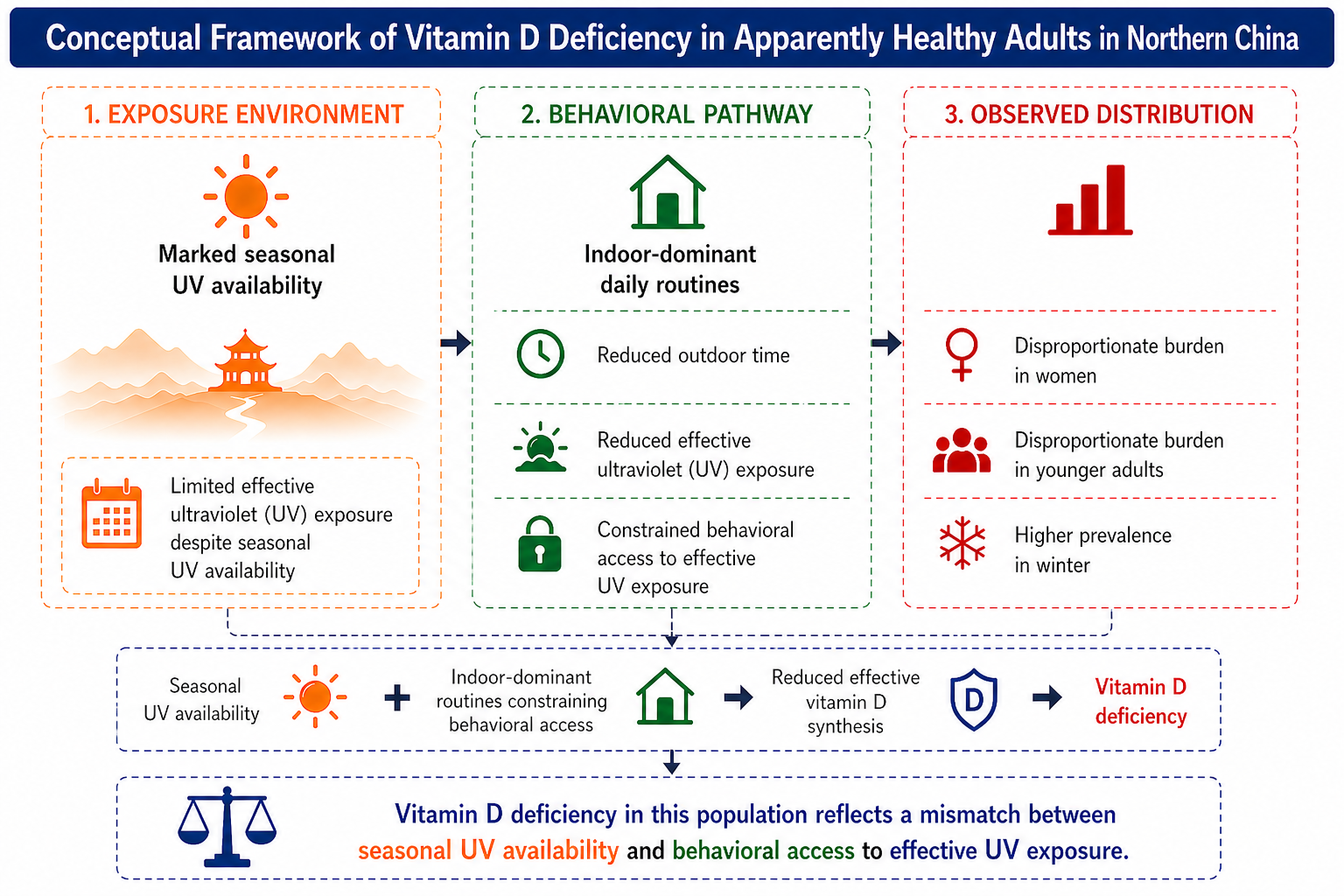

Supplement: Supplementary file 2 [file Image_1.PNG]
